# Association of social determinants of health and their cumulative inequities with risk of hypertension: a population-based study

**DOI:** 10.3389/fcvm.2025.1607604

**Published:** 2025-10-24

**Authors:** Jinhai Shao, Zhichao Sun, Yong Fang, Bowen Song, Zhongyi Shou, Guangli Cao

**Affiliations:** ^1^ Department of Intensive Care Unit, Shangyu People's Hospital of Shaoxing, Shaoxing University, Shaoxing, Zhejiang, China; ^2^ School of Medicine, Shaoxing University, Shaoxing, Zhejiang, China; ^3^ Department of Radiation Oncology, Nanfang Hospital, Southern Medical University, Guangzhou, Guangdong, China; ^4^ Changsha Medical University, Changsha, Hunan, China; ^5^ Department of Cardiology, Shangyu People's Hospital of Shaoxing, Shaoxing University, Shaoxing, Zhejiang, China; ^6^ Department of Emergency Intensive Care Unit, Shaoxing Second Hospital, Shaoxing, Zhejiang, China

**Keywords:** social determinants of health, NHANES, hypertension, health inequities, mental health

## Abstract

**Background:**

Hypertension remains a global public health challenge with significant socioeconomic disparities. While traditional risk factors are well-documented, the cumulative impact of adverse social determinants of health (SDoH) on hypertension risk warrants further investigation.

**Methods:**

We analyzed data from 36,836 NHANES participants (2005–2018), including 15,082 hypertension cases. Eight SDoH indicators across five domains (economic stability, education, healthcare access, neighborhood environment, and social context) were evaluated using survey-weighted multivariable logistic regression. Primary models adjusted for age, sex, and race with subsequent stratified analyses by sex. Sensitivity analyses further adjusted for clinical covariates including BMI, smoking status, and comorbidities. Additionally, mediation analysis was performed to explore whether depression served as a psychosocial mediator in the association between adverse SDoH and hypertension risk.

**Results:**

Five adverse SDoH showed significant associations with hypertension risk: unemployment (AOR = 1.27, 95%CI: 1.17–1.37), low poverty-income ratio (AOR = 1.20, 95%CI: 1.10–1.31), food insecurity (AOR = 1.25, 95%CI: 1.14–1.36), low education level (AOR = 1.09, 95%CI: 1.03–1.17), and government or no insurance (AOR = 1.08, 95%CI: 1.01–1.15). A clear dose-response relationship emerged, with each additional adverse SDoH increasing hypertension risk (1 factor: AOR = 1.19; 5 factors: AOR = 1.46; *P*-trend < 0.0001). Sex differences were notable, with unemployment more strongly associated in men (AOR = 1.39) and low income more impactful in women (AOR = 1.40). Mediation analysis revealed that depression partially mediated the effects of several adverse SDoH on hypertension, accounting for approximately 9%–13% of the total association.

**Conclusion:**

Adverse SDoH were found to be associated with increased hypertension risk in a cross-sectional analysis, with distinct sex-specific and psychosocial pathways. The partial mediation effect of depression suggests that mental health may play a significant role in linking social disadvantage to hypertension, underscoring the importance of integrating psychosocial considerations into hypertension prevention and management.

## 1 Introduction

Hypertension is a major global health concern and a leading risk factor for cardiovascular disease, stroke, and premature mortality (1–3). Despite significant advances in medical management, the prevalence of hypertension continues to rise, particularly among populations experiencing socioeconomic disadvantages (4, 5). Traditionally, hypertension research has primarily focused on biological and behavioral risk factors such as obesity, smoking, excessive salt intake, and physical inactivity (6, 7). However, growing evidence suggests that social determinants of health (SDoH) also play a crucial role in shaping hypertension risk by influencing health behaviors, access to healthcare, and chronic disease development (8, 9).

SDoH encompass a broad range of socioeconomic and environmental factors, including employment status, income level, food security, healthcare access, and housing stability (10, 11). These factors have been implicated in disparities in hypertension prevalence, with individuals facing financial insecurity, lower educational attainment, and inadequate healthcare access being disproportionately affected (12–14). The mechanisms linking adverse SDoH to hypertension are complex and multifaceted, involving chronic psychosocial stress, food insecurity, and heightened exposure to unhealthy living conditions (15–18). Additionally, systemic inequities contribute to disparities in hypertension management, exacerbating health outcomes among socially disadvantaged groups (19).

To address these issues, our study conducted a large-scale analysis examining the associations between multiple SDoH factors and hypertension risk. We explored sex-specific differences in these associations and investigated the cumulative impact of multiple adverse SDoH on hypertension prevalence. Furthermore, sensitivity analyses were performed to assess the robustness of our findings after adjusting for traditional hypertension risk factors. By providing a comprehensive evaluation of the role of SDoH in hypertension, this study aims to inform targeted public health strategies and promote health equity in hypertension prevention and management.

## 2 Methods

### 2.1 Study population

The National Health and Nutrition Examination Survey (NHANES) is a nationally representative program conducted by the National Center for Health Statistics to assess the health and nutritional status of the U.S. population. By employing a complex, multistage probability sampling design, NHANES collects comprehensive data through interviews, physical examinations, and laboratory tests. The dataset provides valuable insights into various health conditions, risk factors, and nutritional trends, making it a widely utilized resource for epidemiological research and public health assessments.

**Figure 1:**
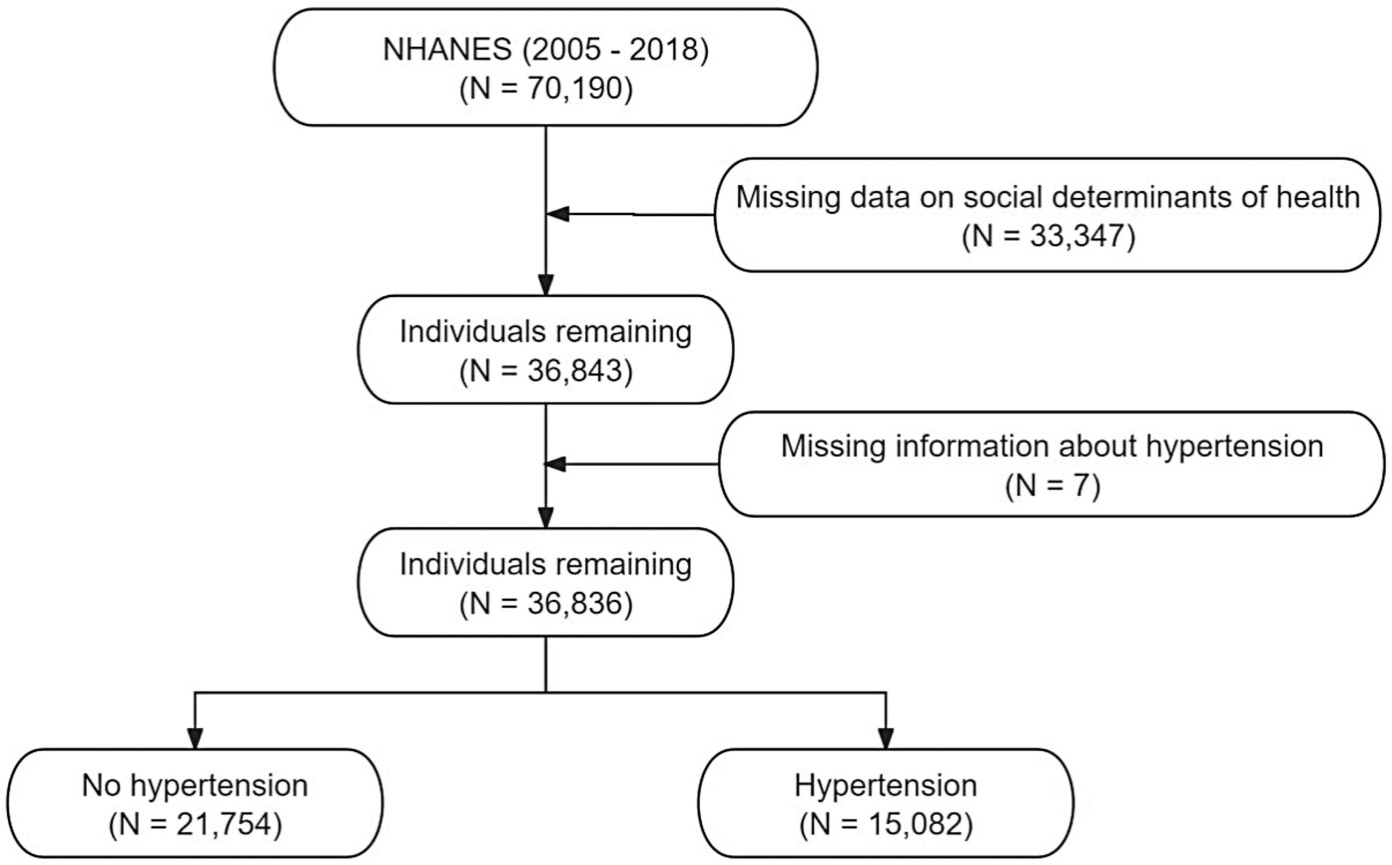
The selection process of NHANES 2005–2018.

A total of 70,190 participants from the NHANES 2005–2018 dataset were initially considered. After excluding 33,347 individuals due to missing data on social determinants of health, 36,843 participants remained. An additional 7 individuals were excluded due to missing hypertension information, resulting in a final analytical sample of 36,836 participants. Among them, 15,082 were classified as having hypertension, while 21,754 were identified as not having hypertension. Figure 1 shows the flow chart of inclusion and exclusion of participants.

### 2.2 Exposure variable

**Figure 2:**
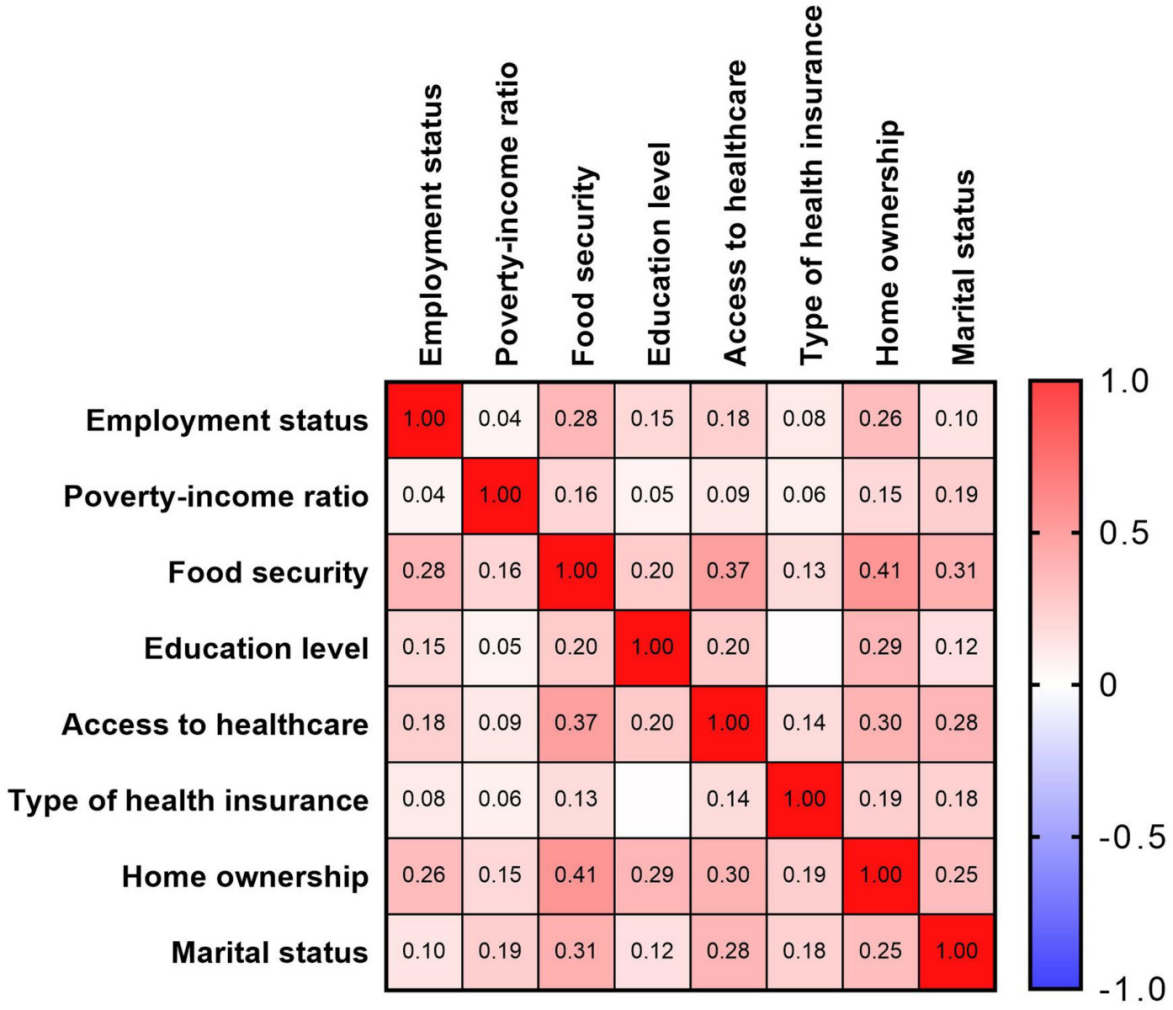
Spearman correlations between eight social determinants of health variables, U.S. NHANES 2005–2018.

This study utilized the Healthy People 2030 framework to define five core domains of SDoH: economic stability, education access and quality, healthcare access and quality, neighborhood and built environment, and social and community context. Corresponding NHANES survey data were used to assess these domains. We selected eight key indicators representing these domains: (1) employment status, (2) poverty-income ratio (PIR), (3) food security, (4) education level, (5) access to healthcare, (6) type of health insurance, (7) home ownership, and (8) marital status. As shown in Figure 2, Spearman correlation analysis demonstrated mild to moderate associations among these SDoH indicators. In addition, to assess multicollinearity, we calculated the Generalized Variance Inflation Factor (GVIF) and its adjusted form (Adjusted GVIF) for each social determinant of health (SDoH) variable. All variables showed GVIF values close to 1, indicating minimal multicollinearity in our regression models (Supplementary Table S1).

To examine the association between cumulative adverse SDoH and hypertension risk, We constructed a cumulative adverse SDoH score following approaches used in prior research (20, 21), where dichotomized indicators are summed to capture the overall burden of social disadvantage. Among the eight SDoH indicators initially considered, five (unemployment, low PIR, food insecurity, low educational attainment, and non-private health insurance) were retained, as they showed statistically significant associations with hypertension in multivariable logistic regression models. The remaining three indicators (access to healthcare, home ownership, and marital status) were not significantly associated and were therefore excluded from the cumulative score. The final cumulative score ranged from 0 to 5, with higher scores indicating greater social disadvantage.

### 2.3 Outcome variable

A standardized blood pressure measurement protocol, consistent with recommendations from the American Heart Association, was employed for data collection between 2007 and 2018. Trained healthcare professionals measured blood pressure using a mercury sphygmomanometer with an appropriately sized cuff. Measurements were taken after the participant remained seated in a resting state for 5 min, with three consecutive readings recorded at 30 s intervals. The average of these three readings was used to determine systolic and diastolic blood pressure levels. Hypertension was defined as meeting any of the following criteria: systolic blood pressure ≥140 mmHg, diastolic blood pressure ≥90 mmHg, self-reported history of hypertension, or current use of antihypertensive medication.

### 2.4 Covariates

This study accounted for several important factors in the analysis. To investigate the link between SDoH and hypertension, age, sex, and race/ethnicity were included as fundamental control variables. Moreover, a range of health-related indicators—such as body mass index (BMI), smoking and drinking habits, participation in physical activities, hyperlipidemia, diabetes, and chronic kidney disease (CKD)—were considered. These indicators were incorporated into sensitivity analyses to assess their potential modifying effect on the relationship between SDoH and hypertension.

### 2.5 Statistical analysis

In our analysis, we accounted for the complex multistage sampling design of NHANES and applied the corresponding survey weights to ensure nationally representative estimates. Following the NHANES Analytic and Reporting Guidelines, we used combined sampling weights (1/7  ×  WTMEC2YR) appropriate for the 14-year survey cycle, which allowed us to adjust for unequal probabilities of selection, nonresponse, and oversampling. Descriptive statistics were calculated to characterize the study population, where continuous variables were expressed as weighted means ± standard errors (SEs), while categorical variables were presented as weighted frequencies and percentages. Group differences in baseline characteristics were assessed using *t*-tests for continuous variables and chi-square tests for categorical variables. To examine the association between individual and cumulative adverse SDoH factors and hypertension risk, survey-weighted logistic regression models were employed. Three models were constructed: an unadjusted model, Model 1 (adjusted for age, sex, and race), and Model 2 (which further adjusted for seven additional SDoH variables). A cumulative SDoH score was created by summing five key adverse SDoH factors, and its impact on stroke risk was evaluated using these regression models. Additionally, a trend analysis was performed to determine whether there was a linear relationship between the cumulative number of adverse SDoH factors and hypertension risk. To further explore potential sex-specific effects of SDoH, stratified analyses by sex were conducted. Sensitivity analyses were also performed by adjusting for potential confounders, including BMI, smoking and alcohol consumption, physical activity levels, hyperlipidemia, diabetes, and CKD, to assess the robustness of the results. Furthermore, mediation analysis was performed to evaluate whether depression partially mediated the relationship between SDoHs and hypertension risk, considering their potential dual roles as outcomes of adverse SDoHs and risk factors for hypertension. All statistical tests were two-sided, with a significance threshold set at *P* < 0.05, and survey weights were applied to ensure representativeness of the findings.

## 3 Result

### 3.1 Demographic characteristics

The study included 36,836 participants, with 40.94% diagnosed with hypertension (Table 1). Significant differences in demographic and socioeconomic characteristics were observed between participants with and without hypertension (*P* < 0.001). Hypertensive participants were more likely to be older, with 37.62% aged 60–79 years and 8.81% aged ≥80 years, compared to younger age groups. Males had a slightly lower prevalence/proportion (49.60%) than females (50.40%). Racial disparities were also evident: among hypertensive participants, 69.96% were White and 13.71% were Black, whereas 5.67% were Mexican American.

**Table 1:** Survey-weighted characteristic variables of the study participants stratified by hypertension.

Characteristic	Estimaate U.S	Total	Hypertension		*P*-value^a^
Variable	Population (*n*)		No	Yes	
Total patients, *n* (%)	208,574,322	36,836	21,754 (59.06)	15,082 (40.94)	
Age, years					**<0** **.** **0001**
20–39	76,292,490	12,286 (36.58)	10,454 (50.21)	1,832 (14.57)	
40–59	77,498,089	11,582 (37.16)	6,831 (36.68)	4,751 (39.00)	
60–79	43,831,039	9,516 (21.01)	2,888 (11.54)	6,628 (37.62)	
≥ 80	8,795,051	2,393 (4.22)	578 (1.57)	1,815 (8.81)	
Sex					**<0** **.** **001**
Male	100,400,941	17,879 (48.14)	10,353 (47.29)	7,526 (49.60)	
Female	108,173,381	18,957 (51.86)	11,401 (52.71)	7,556 (50.40)	
Race					**<0** **.** **0001**
White	141,587,689	15,722 (67.88)	9,053 (66.68)	6,669 (69.96)	
Black	23,395,503	8,072 (11.22)	4,047 (9.77)	4,025 (13.71)	
Mexiacan	16,916,232	5,686 (8.11)	3,880 (9.53)	1,806 (5.67)	
Other	26,674,898	7,356 (12.79)	4,774 (14.03)	2,582 (10.67)	
Employment status					**<0** **.** **0001**
Employed, student, or retired	168,756,683	28,316 (80.91)	16,985 (81.86)	11,331 (79.28)	
Unemployed	39,817,639	8,520 (19.09)	4,769 (18.14)	3,751 (20.72)	
Poverty-income ratio					**<0** **.** **0001**
≥300%	103,176,554	13,276 (49.47)	8,154 (50.70)	5,122 (47.36)	
<300%	105,397,768	23,560 (50.53)	13,600 (49.30)	9,960 (52.64)	
Food security					0.35
Full security	160,204,005	25,483 (76.81)	14,954 (76.61)	10,529 (77.16)	
Marginal, low, or very low security	48,370,317	11,353 (23.19)	6,800 (23.39)	4,553 (22.84)	
Education level					**<0** **.** **0001**
High school graduate or higher	174,707,015	27,363 (83.76)	16,489 (84.83)	10,874 (81.92)	
Less than high school	33,867,307	9,473 (16.24)	5,265 (15.17)	4,208 (18.08)	
Covered by health insurance					**<0** **.** **0001**
Yes	173,849,128	30,248 (83.35)	16,697 (79.20)	13,551 (90.47)	
No	34,725,193	6,588 (16.65)	5,057 (20.80)	1,531 (9.53)	
Type of health insurance					**<0** **.** **0001**
Private	130,940,734	19,246 (62.78)	11,670 (63.98)	7,576 (60.73)	
Government or none	77,633,588	17,590 (37.22)	10,084 (36.02)	7,506 (39.27)	
Home ownership					**<0** **.** **0001**
Own home	141,006,689	22,515 (67.61)	12,474 (64.31)	10,041 (73.26)	
Rent home or other arrangement	67,567,633	14,321 (32.39)	9,280 (35.69)	5,041 (26.74)	
Marital status					0.48
Married or living with a partner	130,962,747	21,336 (62.79)	12,717 (62.60)	8,619 (63.11)	
Not married nor living with a partner	77,611,575	15,500 (37.21)	9,037 (37.40)	6,463 (36.89)	

a The *P*-values were assessed by *t*-test (continuous variables) or by chi-square test (categorical variables) to represent the differences of participants with and without urge urinary incontinence. *P*-values presented with bold valued were statistically significant with *P*-value < 0.05.

Continuous variables are presented as weighted mean ± SE, and categorical variables are presented as counting (*n*) and survey-weighted percentage (%).

NHANES, national health and nutrition examination survey; SDoH, social determinants of health; SE, standard error.

Among hypertensive participants, 20.72% were unemployed, 52.64% had a lower PIR, and 18.08% had less than a high school education, these proportions were higher than in the non-hypertensive group (*p* < 0.001). Additionally, a higher proportion of hypertensive participants had government insurance or were uninsured (39.27%) and were more likely to own their homes (73.26%). Most demographic and socioeconomic characteristics differed between participants with and without hypertension (*p* < 0.001), except marital status and food security. These findings emphasize notable disparities in SDoH factors that may contribute to hypertension risk.

### 3.2 Associations between SDoHs and hypertension

The logistic regression analysis revealed significant associations between multiple SDoH and hypertension risk (Table 2). In the crude model, unemployment, lower PIR, lower education level, government or no insurance, and renting a home were all significantly associated with higher odds of hypertension. However, after adjusting for key covariates in Model 1 (age, sex, and race) and further controlling for other SDoH variables in Model 2, some associations weakened while others remained robust.

**Table 2:** Comparison between different survey-weighted logistic regression models of the relationship between social determinants of health and hypertension.

	Crude model		Model 1		Model 2	
SDoH Variables	COR (95% CI)	*P*—value	AOR (95% CI)	*P*—value	AOR (95% CI)	*P*—value
Employment status						
Employed, student, or retired	Reference		Reference		Reference	
Unemployed	**1.18 (1.11, 1.26)**	**<0**.**0001**	**1.44 (1.34, 1.55)**	**<0**.**0001**	**1.27 (1.17, 1.37)**	**<0.0001**
Poverty-income ratio						
≥300%	Reference		Reference		Reference	
<300%	**1.14 (1.07, 1.22)**	**<0**.**0001**	**1.34 (1.24, 1.45)**	**<0**.**0001**	**1.20 (1.10, 1.31)**	**<0**.**001**
Food security						
Full security	Reference		Reference		Reference	
Marginal, low, or very low security	0.97 (0.91, 1.04)	0.35	**1.40 (1.30, 1.51)**	**<0**.**0001**	**1.25 (1.14, 1.36)**	**<0.0001**
Education level						
High school graduate or higher	Reference		Reference		Reference	
Less than high school	**1.23 (1.15, 1.33)**	**<0**.**0001**	**1.23 (1.13, 1.33)**	**<0**.**0001**	**1.09 (1.03, 1.17)**	**0**.**02**
Access to healthcare						
Yes	Reference		Reference		Reference	
No	**0.40 (0.37, 0.44)**	**<0**.**0001**	**0.66 (0.60, 0.73)**	**<0**.**0001**	**0.60 (0.55, 0.66)**	**<0**.**0001**
Type of health insurance						
Private	Reference		Reference		Reference	
Government or none	**1.15 (1.08, 1.22)**	**<0**.**0001**	**1.25 (1.16, 1.34)**	**<0**.**0001**	**1.08 (1.01, 1.15)**	**0**.**01**
Home ownership						
Own home	Reference		Reference		Reference	
Rent home or other arrangement	**0.66 (0.61, 0.70)**	**<0**.**0001**	**1.12 (1.04, 1.22)**	**0**.**01**	0.99 (0.91, 1.08)	0.83
Marital status						
Married or living with a partner	Reference		Reference		Reference	
Not married nor living with a partner	0.98 (0.92, 1.04)	0.48	**1.12 (1.04, 1.19)**	**0**.**002**	1.06 (0.99, 1.13)	0.08

For each of 8 dichotomized SDoH variables: Crude model was an un-adjusted model. Model 1 was adjusted for age, sex, and race. Model 2 was adjusted for age,sex, race, and other 7 dichotomized SDoH variables. Results of COR (95% CI), AOR (95% CI), *P*-value presented with bold valued were statistically significant with *P*-value < 0.05.

AOR, adjusted odds ratio; CI, confidence interval; COR, crude odds ratio; NHANES, national health and nutrition examination survey; SDoH, social determinants of health.

Unemployment was consistently associated with a higher risk of hypertension, with the adjusted odds ratio (AOR) in Model 2 at 1.27 (95% CI: 1.17–1.37, *P* < 0.0001). Similarly, individuals with a PIR below 300% had an increased hypertension risk (AOR: 1.20, 95% CI: 1.10–1.31, *P* < 0.001). Food insecurity showed no significant association in the crude model, but after adjustment, those experiencing marginal to very low food security had higher odds of hypertension (AOR: 1.25, 95% CI: 1.14–1.36, *P* < 0.0001).

Education level also played a role, as participants with less than a high school education had a higher likelihood of hypertension (AOR: 1.09, 95% CI: 1.03–1.17, *P* = 0.02). Notably, individuals without healthcare access exhibited a lower hypertension risk in all models (AOR: 0.60, 95% CI: 0.55–0.66, *P* < 0.0001). Furthermore, reliance on government or no insurance was associated with an increased risk (AOR: 1.08, 95% CI: 1.01–1.15, *P* = 0.01).

In contrast, homeownership and marital status showed weaker or non-significant associations. While renting was initially linked to lower hypertension risk, the association became non-significant in Model 2 (AOR: 0.99, 95% CI: 0.91–1.08, *P* = 0.83). Similarly, not being married was only marginally associated with hypertension in Model 1 (AOR: 1.12, *P* = 0.002) but lost significance in Model 2 (AOR: 1.06, *P* = 0.08).

**Figure 3:**
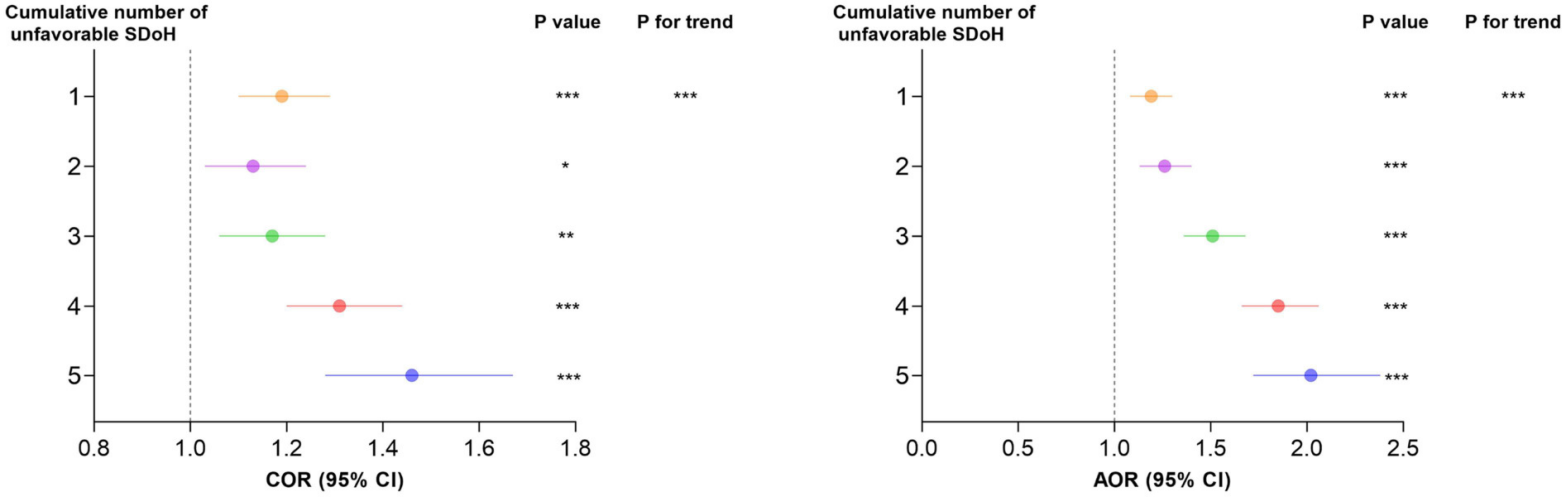
Comparison between different survey-weighted logistic regression models of the weighted relationship between cumulative number of unfavorable SDoH and hypertension. Cumulative unfavorable SDoH score was calculated by summing five dichotomized SDoH (unemployment, low PIR, food insecurity, low educational level, and non-private insurance), coded as 0 = favorable, 1 = unfavorable. Results of COR (95% CI) are based on unadjusted models. Adjusted odds ratios (AOR, 95% CI) are based on models adjusted for age, sex, and race. The reference category is participants with 0 unfavorable SDoH. *P*-values for trend represent the significance of the linear association across increasing number of unfavorable SDoH. * *p* < 0.05, ** *p* < 0.01, *** *p* < 0.001. AOR, adjusted odds ratio; CI, confidence interval; COR, crude odds ratio; SDoH, social determinants of health.

Figure 3 shows a clear dose-response association between cumulative unfavorable SDoH and hypertension risk. The odds ratios increased progressively with each additional unfavorable SDoH: 1.19 (1.10–1.29) for 1 factor, 1.13 (1.03–1.24) for 2, 1.17 (1.06–1.28) for 3, 1.31 (1.20–1.44) for 4, and 1.46 (1.28–1.67) for 5 factors. After adjusting for age, sex, and race, the association between accumulation of adverse SDoH and the risk of hypertension remained consistent. Notably, in both models, a gradual increase in the risk of hypertension was observed as the number of adverse SDoH increased (*P* for trend < 0.0001).

### 3.3 Subgroup analysis

Our findings revealed significant sex differences in the association between SDoH and hypertension (Table 3). While unemployment increased hypertension risk in both sexes, the effect was more pronounced in males (AOR = 1.39, 95%CI: 1.22–1.58) than females (AOR = 1.23, 95%CI: 1.11–1.37). Similarly, low PIR (<300%) elevated hypertension risk across sexes, though the association was stronger in females (AOR = 1.40, 95%CI: 1.23–1.59) compared to males (AOR = 1.03, 95%CI: 0.92–1.16).

**Table 3:** Association between social determinants of health and odds of hypertension in survey-weighted logistic regression models stratified by sex groups, U.S. NHANES 2005–2018.

Characteristic	Female		Male		*P* for interaction
Variable	COR (95% CI)	AOR (95% CI)	COR (95% CI)	AOR (95% CI)	
Employment status					**0**.**0054**
Employed, student, or retired	Reference	Reference	Reference	Reference	
Unemployed	**1.10 (1.01, 1.19)** *	**1.23 (1.11, 1.37)** ^***^	**1.40 (1.27, 1.54)** ^***^	**1.39 (1.22, 1.58)** ^***^	
Poverty-income ratio					**<0.0001**
≥300%	Reference	Reference	Reference	Reference	
<300%	**1.39 (1.26, 1.52)** ^***^	**1.40 (1.23, 1.59)** ^***^	0.94 (0.87, 1.02)	1.03 (0.92, 1.16)	
Food security					0.5506
Full security	Reference	Reference	Reference	Reference	
Marginal, low, or very low security	1.01 (0.93, 1.10)	**1.22 (1.09, 1.36)** ^***^	0.93 (0.84, 1.02)	**1.28 (1.13, 1.45)** ^***^	
Education level					**<0.0001**
High school graduate or higher	Reference	Reference	Reference	Reference	
Less than high school	**1.55 (1.40, 1.71)** ^***^	**1.24 (1.09, 1.41)** **	0.98 (0.89, 1.08)	0.95 (0.85, 1.07)	
Covered by health insurance					0.3975
Yes	Reference	Reference	Reference	Reference	
No	**0.41 (0.35, 0.47)** ^***^	**0.62 (0.52, 0.74)** ^***^	**0.37 (0.34, 0.42)** ^***^	**0.58 (0.52, 0.66)** ^***^	
Type of health insurance					**0**.**0049**
Private	Reference	Reference	Reference	Reference	
Government or none	**1.27 (1.17, 1.38)** ^***^	**1.12 (1.00, 1.25)** *	1.03 (0.95, 1.12)	1.06 (0.95, 1.19)	
Home ownership					0.7798
Own home	Reference	Reference	Reference	Reference	
Rent home or other arrangement	**0.69 (0.62, 0.75)** ^***^	0.98 (0.87, 1.09)	**0.63 (0.58, 0.69)** ^***^	1.00 (0.90, 1.12)	
Marital status					**<0.0001**
Married or living with a partner	Reference	Reference	Reference	Reference	
Not married nor living with a partner	**1.28 (1.18, 1.39)** ^***^	1.04 (0.94, 1.14)	**0.73 (0.67, 0.79)** ^***^	0.97 (0.88, 1.08)	

* *p* < 0.05.

** *p* < 0.01.

*** *p* < 0.001.

For all SDoH variables: Crude model was unadjusted. For each of eight dichotomized SDoH variables: Multivariable models refer to Model 2 which adjusted for age, sex, race, and other seven dichotomized SDoH variables. Results of AOR (95% CI), *p* for trend and *p*-Value presented with bold valued were statistically significant with *p* < 0.05.

AOR, adjusted odds ratio; CI, confidence interval; SDoH, social determinants of health.

Distinct patterns emerged for other SDoH factors. Lower education significantly predicted hypertension only in females (AOR = 1.24, 95%CI: 1.09–1.41), while showing no effect in males (AOR = 0.95, 95%CI: 0.85–1.07). Conversely, government or no insurance demonstrated a stronger association in females (AOR = 1.12, 95%CI: 1.00–1.25) than males (AOR = 1.06, 95%CI: 0.95–1.19). Marital status showed particularly divergent effects, with unmarried women exhibiting higher hypertension risk (AOR = 1.04, 95%CI: 0.94–1.14) while unmarried men showed a potential protective trend (AOR = 0.97, 95%CI: 0.88–1.08).

**Figure 4:**
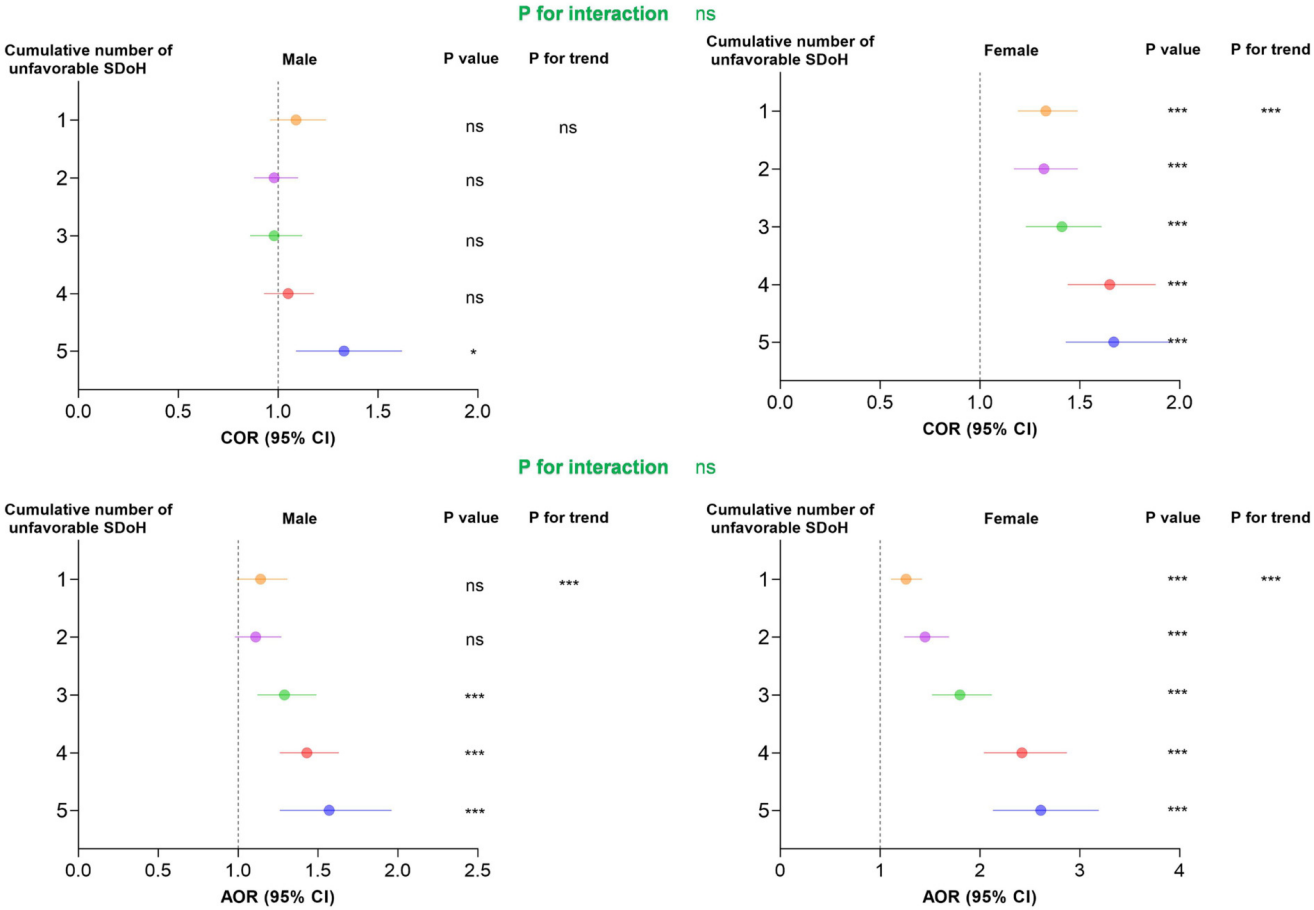
Association between cumulative number of unfavorable SDoH associated with odds of hypertension in survey-weighted logistic regression models stratified by sex, U.S. NHANES 2005−2018. Cumulative unfavorable SDoH score was calculated by summing five dichotomized SDoH (unemployment, low PIR, food insecurity, low educational level, and non-private insurance), coded as 0 = favorable, 1 = unfavorable. Results of COR (95% CI) are based on unadjusted models. Adjusted odds ratios (AOR, 95% CI) are based on models adjusted for age and race. The reference category is participants with 0 unfavorable SDoH. *P*-values for trend represent the significance of the linear association across increasing number of unfavorable SDoH. * *p* < 0.05, ** *p* < 0.01, *** *p* < 0.001. AOR, adjusted odds ratio; CI, confidence interval; COR, crude odds ratio; SDoH, social determinants of health.

Notably, as shown in Figure 4, the cumulative burden of unfavorable SDoH exhibited a dose-response relationship with hypertension risk in both sexes (*P* for trend < 0.001). However, the risk gradient appeared steeper in females than males at higher SDoH counts, suggesting women may be particularly vulnerable to the compounding effects of multiple social disadvantages.

### 3.4 Sensitivity analysis

To validate the robustness of our findings, we performed comprehensive sensitivity analyses by additionally adjusting for potential confounding factors including BMI, smoking status, alcohol consumption, recreational activity, hyperlipidemia, diabetes, and CKD (Table 4). In the fully adjusted model, all key unfavorable SDoH maintained significant associations with hypertension risk: unemployment (AOR = 1.20, 95% CI: 1.10–1.30), low PIR (AOR = 1.06, 95% CI:1.01–1.17), food insecurity (AOR = 1.11, 95% CI: 1.00–1.23), low educational attainment (AOR = 1.08, 95% CI: 1.03–1.19), and government or no insurance (AOR = 1.02, 95% CI: 1.00–1.07).

**Table 4:** Further adjustments in sensitivity analyses for individual social determinants of health (sDoH) associated with odds of hypertension by survey-weighted logistic regression models, U.S.

SDoH Variables	Adjusted for BMI, smoking, drinking, and recreational activity, AOR (95% CI)	Adjusted for hyperlipidemia, diabetes, and CKD, AOR (95% CI)	Adjusted for BMI, smoking, drinking, recreational activity, hyperlipidemia, diabetes, and CKD, AOR (95% CI)
Employment status			
Employed, student, or retired	Reference	Reference	Reference
Unemployed	**1.24 (1.14, 1.35)** ^***^	**1.20 (1.11, 1.30)** ^***^	**1.20 (1.10, 1.30)** ^***^
Poverty-income ratio			
≥300%	Reference	Reference	Reference
<300%	**1.11 (1.02, 1.22)** ^*^	**1.13 (1.03, 1.25)** ^*^	**1.06 (1.01, 1.17)** ^*^
Food security			
Full security	Reference	Reference	Reference
Marginal, low, or very low security	**1.11 (1.01, 1.22)** ^*^	**1.20 (1.09, 1.32)** ^**^	**1.11 (1.00, 1.23)** ^*^
Education level			
High school graduate or higher	Reference	Reference	Reference
Less than high school	**1.08 (1.01, 1.17)** ^*^	**1.02 (0.93, 1.11)**	**1.08 (1.03, 1.19)** ^*^
Covered by health insurance			
Yes	Reference	Reference	Reference
No	**0.67 (0.60, 0.74)** ^**^	**0.66 (0.60, 0.73)** ^***^	**0.70 (0.63, 0.78)** ^***^
Type of health insurance			
Private	Reference	Reference	Reference
Government or none	1.05 (0.97, 1.14)	**1.08 (1.05, 1.13)** ^*^	**1.02 (1.00, 1.07)** ^*^
Home ownership			
Own home	Reference	Reference	Reference
Rent home or other arrangement	0.98 (0.90, 1.08)	0.98 (0.90, 1.07)	0.98 (0.89, 1.08)
Marital status			
Married or living with a partner	Reference	Reference	Reference
Not married nor living with a partner	**1.08 (1.00, 1.16)** ^*^	1.06 (0.98, 1.14)	1.07(0.99, 1.16)

* *p* < 0.05.

** *p* < 0.01.

*** *p* < 0.001.

For each of 8 dichotomized SDoH variables: Sensitivity analysis was performed after adjusting for age, sex, race, and other 7 dichotomized SDoH variables to further adjust for other confounding factors.

Results of AOR (95% CI), *P*-value presented with bold valued were statistically significant with *P*-value < 0.05.

AOR, adjusted odds ratio; CI, confidence interval; NHANES, national health and nutrition examination survey; SDoH, social determinants of health.

**Figure 5:**
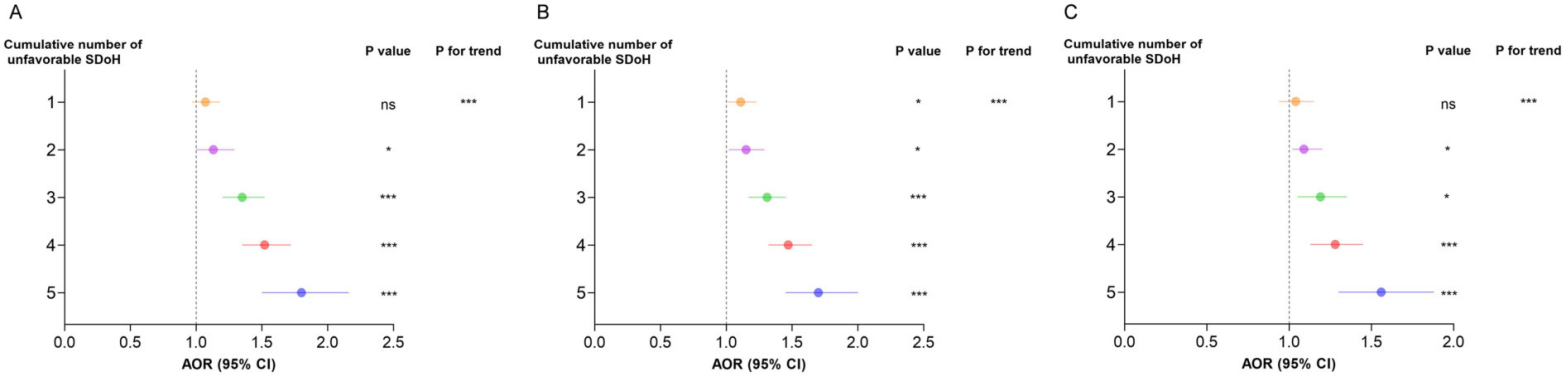
Further adjustments in sensitivity analyses for cumulative number of unfavorable SDoH associated with odds of hypertension by survey-weighted logistic regression models, U.S. NHANES 2005−2018. **(A)** Sensitivity analysis is based on Model 2 to further adjust BMI, smoking, drinking, and recreational activity. **(B)** Sensitivity analysis is based on Model 2 to further adjust hypertension, diabetes, CVD, CKD, and depression. **(C)** Sensitivity analysis is based on Model 2 to further adjust BMI, smoking, drinking, recreational activity, hypertension, diabetes, CVD, CKD, and depression. Cumulative unfavorable SDoH score was calculated by summing five dichotomized SDoH (unemployment, low PIR, food insecurity, low educational level, and non-private insurance), coded as 0 = favorable, 1 = unfavorable. Results of COR (95% CI) are based on unadjusted models. Adjusted odds ratios (AOR, 95% CI) are based on models adjusted for age, sex, and race. The reference category is participants with 0 unfavorable SDoH. *P*-values for trend represent the significance of the linear association across increasing number of unfavorable SDoH. *p < 0.05, **p < 0.01, ***p < 0.001. AOR, adjusted odds ratio; CI, confidence interval; SDoH, social determinants of health.

Furthermore, our analysis revealed a significant dose-response association between cumulative unfavorable SDoH and hypertension risk (Figure 5). Compared to individuals with no unfavorable SDoH, those with increasing numbers of unfavorable SDoH showed progressively higher hypertension risk: 1 SDoH (AOR = 1.04, 95% CI: 0.94–1.15), 2 SDoH (AOR = 1.09, 95% CI: 1.02–1.20), 3 SDoH (AOR = 1.19, 95% CI: 1.05–1.35), 4 SDoH (AOR = 1.28, 95% CI: 1.13–1.45), and 5 SDoH (AOR = 1.56, 95% CI:1.30–1.88). The trend test for this graded association was highly significant (*P* for trend < 0.001), demonstrating that the accumulation of multiple unfavorable SDoH substantially elevates hypertension risk in a dose-dependent manner.

### 3.5 Mediation analyses

**Figure 6:**
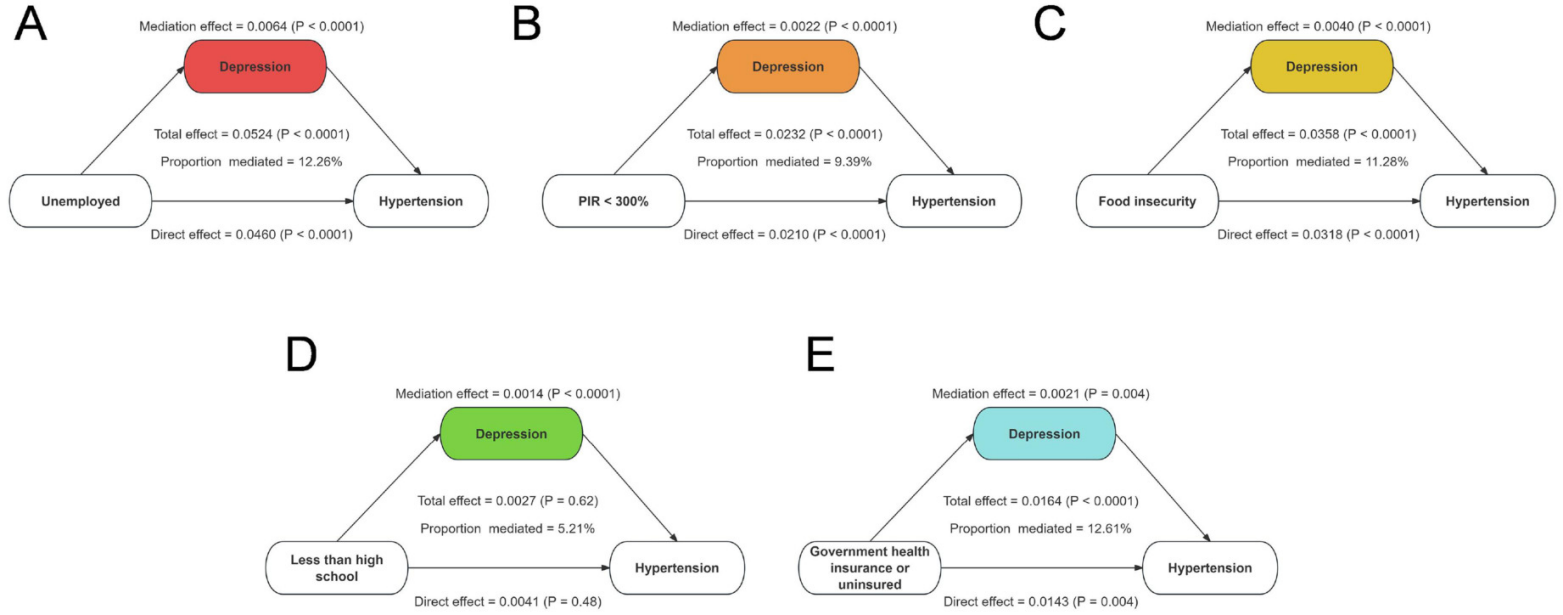
Mediation effects of depression between unfavorable SDoH and odds of hypertension. **(A)** Proportion of unemployed and hypertension risk mediated by depression. **(B)** Proportion of low PIR and hypertension risk mediated by depression. **(C)** Proportion of food insecurity and hypertension risk mediated by depression. **(D)** Proportion of less than high school and hypertension risk mediated by depression. **(E)** Proportion of government health insurance or uninsured and hypertension risk mediated by depression.

Given that adverse SDoH are associated with an increased risk of depression, we conducted a mediation analysis to examine whether depression partially mediated the relationship between adverse SDoH and hypertension. As shown in Figure 6, depression significantly mediated the associations between several SDoH and hypertension risk. Specifically, depression accounted for 12.26% of the effect of unemployment, 9.39% of the effect of low PIR, 11.28% of the effect of food insecurity, and 12.61% of the effect of government or no insurance on hypertension risk. Notably, the mediating role of depression in the association between low educational attainment and hypertension risk was not significant (*P* > 0.05).

## 4 Discussion

This national study reveals that adverse social determinants of health (SDoH) significantly influence hypertension risk through both independent and cumulative effects. Key socioeconomic factors—particularly unemployment, low PIR, food insecurity, low education, and non-private insurance—demonstrated robust associations with hypertension prevalence after demographic adjustment. Notably, we identified a graded increase in hypertension risk with accumulating adverse SDoH. This study highlights the critical role of structural social inequities in shaping hypertension disparities across population groups.

Hypertension is a major global health concern and a leading risk factor for cardiovascular diseases, including stroke, heart failure, and CKD (22–24). Despite advancements in medical treatment, its prevalence remains high, particularly among socially and economically disadvantaged populations (25, 26). Previous studies have established that SDoH significantly contribute to disparities in hypertension risk (27–29). Our study highlights the significant role of SDoH in shaping hypertension risk, revealing both independent effects of specific adverse SDoH factors and a cumulative burden associated with multiple social disadvantages. Unemployment, low PIR, food insecurity, low education level, and lack of private health insurance were all significantly associated with an increased risk of hypertension, even after adjusting for conventional risk factors such as BMI, smoking, physical activity, hyperlipidemia, diabetes, and CKD. Notably, a clear dose-response relationship was observed, wherein individuals with a greater number of adverse SDoH factors exhibited a progressively higher risk of hypertension. These findings underscore the importance of addressing structural inequities that contribute to cardiovascular disease risk, emphasizing the need for a broader public health approach beyond traditional medical and lifestyle interventions.

Emerging evidence suggests that the impact of SDoH outcomes varies by sex, with men and women experiencing different vulnerabilities to socioeconomic stressors (30, 31). For instance, financial insecurity and employment instability have been linked to adverse cardiovascular outcomes, though the mechanisms may differ between sexes (32–34). Sex-specific analyses revealed distinct patterns in how SDoH factors influence hypertension risk. While unemployment and low income were strong predictors of hypertension in both sexes, the effects were more pronounced in males, potentially reflecting greater financial stress and occupational instability. Conversely, lower education levels and reliance on non-private health insurance were more strongly associated with hypertension in females, suggesting that women may face unique barriers to healthcare access and chronic disease management. The cumulative burden of multiple adverse SDoH factors had a steeper impact on hypertension risk in females, indicating that women may be more vulnerable to the compounded effects of social disadvantage. Beyond these statistical associations, broader contextual factors may further explain the observed sex-specific differences. Women often face compounded disadvantages due to caregiving responsibilities, lower lifetime income, and reduced access to healthcare resources, which can amplify the adverse impact of low income, low education, and reliance on non-private insurance. Additionally, psychosocial stressors such as gender-based discrimination and limited social support may exacerbate cardiovascular vulnerability among women. These findings highlight the necessity of implementing targeted, sex-specific public health interventions to address disparities in hypertension risk.

From a policy and intervention perspective, our results suggest that reducing hypertension disparities requires integrating social and economic strategies into traditional cardiovascular risk reduction efforts. Expanding economic opportunities, improving educational access, and ensuring affordable healthcare coverage could be essential measures to mitigate the effects of adverse SDoH on hypertension risk. Given the strong association between cumulative SDoH burden and hypertension, future research should focus on longitudinal studies to better understand the causal mechanisms linking SDoH factors to hypertension and evaluate the effectiveness of policy-driven interventions in reducing social inequities in cardiovascular health.

Moreover, it is important to acknowledge that unmeasured psychosocial and environmental factors may also influence the observed associations. Variables such as perceived stress, experiences of discrimination, social support, and neighborhood environmental exposures are known to affect both social determinants of health and hypertension outcomes (35–37). Although these factors were not available in the NHANES dataset, their omission may result in residual confounding and partially explain the associations identified in this study. To partially address this limitation, we incorporated a mediation analysis using depression, an established psychosocial indicator, and found that it significantly mediated the relationship between several adverse SDoH and hypertension. Future research integrating a broader spectrum of psychosocial and environmental measures is warranted to provide a more comprehensive understanding of the complex pathways linking social disadvantage to hypertension.

This study benefits from its large, nationally representative sample and comprehensive evaluation of multiple SDoH domains using standardized NHANES protocols. The examination of cumulative SDoH effects and sex-specific patterns provides novel insights into hypertension disparities. However, several limitations should be noted. The cross-sectional design precludes causal inference, and residual confounding may persist despite multivariable adjustment. While we included major SDoH indicators, some potentially important social factors (e.g., discrimination, social support) were not assessed. Additionally, the observational nature of NHANES data limits generalizability to non-U.S. populations. Future longitudinal studies incorporating more granular SDoH measures are needed to confirm these findings and elucidate underlying mechanisms.

## 5 Conclusion

This national study demonstrates that adverse social determinants of health, particularly unemployment, low PIR, food insecurity, limited education, and government or no insurance, are independently and cumulatively associated with increased hypertension risk. Moreover, depression was found to partially mediate these associations, suggesting that psychosocial pathways may play an important role in linking social disadvantage to hypertension. These findings highlight the need for integrated prevention strategies that address both socioeconomic inequities and mental health factors. Future research should employ longitudinal designs to clarify causal mechanisms and evaluate targeted, multidimensional interventions aimed at mitigating the social and psychological burden of hypertension among high-risk populations.

## Data Availability

Publicly available datasets were analyzed in this study. This data can be found here: https://www.cdc.gov/nchs/nhanes/?CDC_AAref_Val=https://www.cdc.gov/nchs/nhanes/index.htm
