# Supplementary material for: Association of social determinants of health and their cumulative inequities with risk of hypertension: a population-based study
Source: Front Cardiovasc Med. 2025 Oct 24;12:1607604. doi: 10.3389/fcvm.2025.1607604 (PMC12592167; doi:10.3389/fcvm.2025.1607604)
Supplement: Supplementary file 1 [file Table1.docx]

**Supplementary Table 1 Variance Inflation Factors (VIF) and Adjusted VIF for SDoH Variables**

| **SDoH Variable** | **GVIF** | **Degrees of Freedom (Df)** | **Adjusted GVIF (GVIF^1/(2*Df)^)** |
| --- | --- | --- | --- |
| Employment status | 1.162741 | 1 | 1.078305 |
| Poverty-income ratio | 1.431818 | 1 | 1.196586 |
| Food security | 1.279107 | 1 | 1.130976 |
| Education level | 1.232595 | 1 | 1.110223 |
| Access to healthcare | 1.159294 | 1 | 1.076705 |
| Type of health insurance | 1.394356 | 1 | 1.180828 |
| Home ownership | 1.295047 | 1 | 1.138001 |
| Marital status | 1.109157 | 1 | 1.053165 |

Footnote: GVIF values represent the Generalized Variance Inflation Factor for each Social Determinant of Health variable. The adjusted GVIF is calculated as GVIF^1/(2×Df)^, where Df is the degrees of freedom associated with each variable. Values above 1 indicate some degree of multicollinearity, but all variables in this analysis show GVIF values that suggest minimal multicollinearity.
